# A Novel Prognostic Model Based on Ferroptosis-Related Gene Signature for Bladder Cancer

**DOI:** 10.3389/fonc.2021.686044

**Published:** 2021-08-06

**Authors:** Libo Yang, Chunyan Li, Yang Qin, Guoying Zhang, Bin Zhao, Ziyuan Wang, Youguang Huang, Yong Yang

**Affiliations:** ^1^Department of Urology, The Third Affiliated Hospital of Kunming Medical University, Kunming, China; ^2^Second Department of Head and Neck Surgery, The Third Affiliated Hospital of Kunming Medical University, Kunming, China; ^3^Department of Yunnan Tumor Research Institute, The Third Affiliated Hospital of Kunming Medical University, Kunming, China

**Keywords:** ferroptosis, prognosis, biomarkers, bladder cancer, bladder urothelial carcinoma

## Abstract

**Background:**

Bladder cancer (BC) is a molecular heterogeneous malignant tumor; the treatment strategies for advanced-stage patients were limited. Therefore, it is vital for improving the clinical outcome of BC patients to identify key biomarkers affecting prognosis. Ferroptosis is a newly discovered programmed cell death and plays a crucial role in the occurrence and progression of tumors. Ferroptosis-related genes (FRGs) can be promising candidate biomarkers in BC. The objective of our study was to construct a prognostic model to improve the prognosis prediction of BC.

**Methods:**

The mRNA expression profiles and corresponding clinical data of bladder urothelial carcinoma (BLCA) patients were downloaded from The Cancer Genome Atlas (TCGA) and Gene Expression Omnibus (GEO) databases. FRGs were identified by downloading data from FerrDb. Differential analysis was performed to identify differentially expressed genes (DEGs) related to ferroptosis. Univariate and multivariate Cox regression analyses were conducted to establish a prognostic model in the TCGA cohort. BLCA patients from the GEO cohort were used for validation. Gene ontology (GO), Kyoto Encyclopedia of Genes and Genomes (KEGG), and single-sample gene set enrichment analysis (ssGSEA) were used to explore underlying mechanisms.

**Results:**

Nine genes (*ALB, BID, FADS2, FANCD2, IFNG, MIOX, PLIN4, SCD*, and *SLC2A3*) were identified to construct a prognostic model. Patients were classified into high-risk and low-risk groups according to the signature-based risk score. Receiver operating characteristic (ROC) and Kaplan–Meier (K–M) survival analysis confirmed the superior predictive performance of the novel survival model based on the nine-FRG signature. Multivariate Cox regression analyses showed that risk score was an independent risk factor associated with overall survival (OS). GO and KEGG enrichment analysis indicated that apart from ferroptosis-related pathways, immune-related pathways were significantly enriched. ssGSEA analysis indicated that the immune status was different between the two risk groups.

**Conclusion:**

The results of our study indicated that a novel prognostic model based on the nine-FRG signature can be used for prognostic prediction in BC patients. FRGs are potential prognostic biomarkers and therapeutic targets.

## Introduction

Bladder cancer is one of the leading causes of cancer-related death worldwide. As the second most frequent genitourinary malignancy, BC is the 10th most common cancer globally according to global cancer data, with 573,278 new cases diagnosed and 212,526 deaths in 2020 according to Globocan prediction (1). The incidence and mortality of BC have been continuing to increase. Urothelial carcinoma is the most common histologic type, accounting for approximately 90% of primary BC (2). Among the newly diagnosed BC, non-muscular invasive bladder cancer (NMIBC) accounts for approximately 70% and transurethral resection of bladder tumor (TURBT) is the main treatment (3, 4). About 63.24% and 11.76% of the NMIBC patients after TURBT have tumor recurrence and progression (5). Likewise, nearly 50% of muscular invasive bladder cancer (MIBC) patients undergoing radical cystectomy still have local recurrence or distant metastasis, with a 5-year survival rate of only 66% (6). Furthermore, for 30 years, clinicians were stuck with the same, limited range of therapeutics to offer patients with BC, and 5-year survival rates were flat (7). Onset of BC is a complex process, which a multi-factor, multi-step, and multi-gene participation in (8). Therefore, a better understanding of the molecular characterization involved in tumorigenesis and the identification of novel prognostic biomarkers are essential for improving the clinical outcome of patients. The complex etiologic factors, along with the high-level heterogeneity of BC (9), make the prognosis significantly different and prognostic prediction challenging. This calls for the development of novel prognostic models.

Ferroptosis is a newly discovered iron-dependent form of regulated cell death (RCD) that is driven by the lethal accumulation of lipid peroxidation (10, 11). In 2012, it was firstly described that ferroptosis differs from apoptosis, necrosis, and autophagy in terms of morphology, biochemistry, and genetics. Ferroptosis is characterized by the rupture and blistering of cell membranes, mitochondrial shrinkage, increased membrane density, decreased or disappearance of mitochondrial ridges, rupture of outer mitochondrial membranes, and normal-sized nuclei without condensed chromatin (10). Studies have demonstrated strong association of ferroptosis with mammalian neurodegenerative diseases, cancer, and stroke (12). Since the first demonstration in 2012, ferroptosis has received widespread attention as a potential therapeutic pathway for cancer treatment. In recent years, the induction of ferroptosis has emerged as a promising therapeutic alternative to trigger cancer cell death, especially for malignancies that are resistant to traditional treatments (13, 14). Various studies have determined the key role of ferroptosis in killing tumor cells and inhibiting tumor growth (15, 16). A large number of studies demonstrated the potential clinical value of utilizing these deregulated proteins as prognostic biomarkers of malignancy (17–19). Some previous studies have also confirmed the important significance of ferroptosis for the treatment of bladder cancer (20, 21), However, whether these ferroptosis-related genes (FRGs) are correlated with BC patient prognosis remains unclear.

The objective of this study was to determine the prognostic value of FRGs in BC. mRNA expression profiles and corresponding clinical data of bladder urothelial carcinoma (BLCA) were extracted from the public databases. Ferroptosis-related differentially expressed genes (DEGs) closely associated with the prognosis were identified to construct predictive models for the prognosis of BLCA in the TCGA cohort. Then, we validated it in the GEO cohort. Besides this, functional enrichment analysis was performed to explore the underlying mechanisms.

## Materials And Methods

### Data Acquisition TCGA Cohort and GEO Cohort

All datasets used in this study were available to the public. Therefore, ethical approval for this study was not required. This study followed the policies and guidelines for data access and publication specified by the TCGA and GEO databases. Data cutoff was January 20, 2020.

Patients who met the following selection criteria were included: (a) histologically diagnosed with transitional cell carcinoma; (b) available gene expression data; and (c) available survival information. Patients with no complete clinical information were excluded. The RNA sequencing (RNA-seq) dataset and corresponding clinical information of 430 BCLA patients were downloaded from GDC (https://cancergenome.nih.gov/) as training cohort. The gene expression profile was standardized using the variance stabilizing transformation method provided by the “DEseq2” R package. Gene expression annotation information was obtained from the Ensembl website (https://asia.ensembl.org/index.html/). Similarly, the other RNA sequencing (RNA-seq) dataset and corresponding clinical information of 165 BCLA patients were downloaded from the Gene Expression Omnibus database portal website (https://www.ncbi.nlm.nih.gov/geo/) as a validation cohort. Internal standardization was performed *via* the “limma” package. Gene sequencing data annotation was performed with the R package “illuminaHumanv2 GPL6102 platform” from Bioconductor. Then, difference analysis was performed *via* the “Deseq2” R package.

### FRGs and Immune-Related Data

The list of FRGs was download from the FerrDb web portal (http://www.zhounan.org/ferrdb), which contains six datasets. A total of 259 FRGs were identified with the following criteria: driver, suppressor, and marker of ferroptosis. We provided the list in **Supplementary Table S1**. The immune-related data were obtained from the ImmPort web portal (https://immport.org/shared/home).

### Establishment and Validation of a Prognostic Model of FRGs Signature

DEGs related to ferroptosis in tumor tissues and adjacent normal tissues in the TCGA cohort were selected using the “Deseq2” R package, with an absolute log2-fold change (FC) ≥ 1 and an adjusted *p*-value < 0.05. The Venn diagram and heatmap were drawn using the Venn diagrams analysis online website (http://bioinformatics.psb.ugent.be/webtools/Venn/) and the “heatmap” R package. An interaction network for the candidate prognostic DEGs was generated by the online STRING database (version 3.7.1). Plots in the present study were drawn by ggplot2.

FRGs associated with overall survival (OS) were identified with univariate Cox regression analysis. P values were adjusted by Benjamini and Hochberg (BH) method. Multivariate Cox regression model analysis was performed to identify covariates with independent prognostic values for patient survival. Based on a multivariate Cox regression for these genes, a prognostic gene signature using ferroptosis-related DEGs was established.

To reflect the comprehensive effects of the ferroptosis, a risk score of each patient was calculated according to the normalized expression level of each gene and its corresponding regression coefficients. The formula was established as follows: risk score = ∑ (Coefi * Expi). The optimal cutoff values for gene expression were determined using the “surv-cutpoint” function of the “Surviminer” package in R. Patients in TCGA training and GEO validation cohorts were divided into high-risk and low-risk groups based on the median risk score as the cutoff value. Kaplan–Meier survival analysis and log-rank test were used to compare difference in the OS between the stratified groups. Then, receiver operating characteristic (ROC) curve analysis and the area under the ROC curve (AUC) was applied to test the predictive power of the prognostic risk score model. The difference in gene expression between tumor tissues and normal tissues and its correlation with prognosis was further validated by the GEPIA online database (http://gepia.cancer-pku.cn/).

### Construction and Evaluation of a Predictive Nomogram

During the quantification of the risk on individuals in a clinical setting with the integration of multiple risk factors, the nomogram was an excellent tool in the assessment. The independent predictive factors identified by multivariate Cox regression were integrated to construct a predictive nomogram and corresponding calibration curves using the “rms” R package. The closer the calibration curve is to the 45° line, which represents the best prediction, the better is the prognostic prediction performance of the nomogram.

### Function Enrichment Analysis

We applied the “limma” R package to analyze the correlations of DEGs between the high-risk and low-risk groups in TCGA and GEO cohorts, respectively. Gene ontology (GO) and Kyoto Encyclopedia of Genes and Genomes (KEGG) enrichment analysis for DEGs were conducted using the “clusterProfiler” package in R. For differential infiltrating score analysis between the high- and low-risk groups, infiltrating scores of 16 immune cells and 13 immune-related pathways were calculated by single-sample gene set enrichment analysis (ssGSEA) using the “gsva” package in R. The genes related to immune cell infiltration are provided in **Supplementary Table S2**.

### Statistical Analysis

Statistical analyses were carried out with the R software (Version 3.5.3). The Student’s *t*-test was used to compare the gene expression between tumor tissues and adjacent normal tissues. Patients in TCGA training and GEO validation cohorts were divided into high-risk and low-risk groups based on the median risk score. Chi-square test was adopted to compare differences in age, gender, T stage, N (lymph node metastasis) status, M (tumor metastasis) status, diagnosis subtype, and histologic grade between the high- and low-risk groups. Mann–Whitney test with *p*-values adjusted by the BH method was used to compare the ssGSEA scores of immune cells or pathways between the high- and low-risk groups. Kaplan–Meier survival analysis and log-rank test were used to compare difference in the OS between the stratified groups. Univariate and multivariate Cox regression analyses were used to determine independent prognostic factors. *p* < 0.05 was considered statistically significant.

## Results

**Figure 1:**
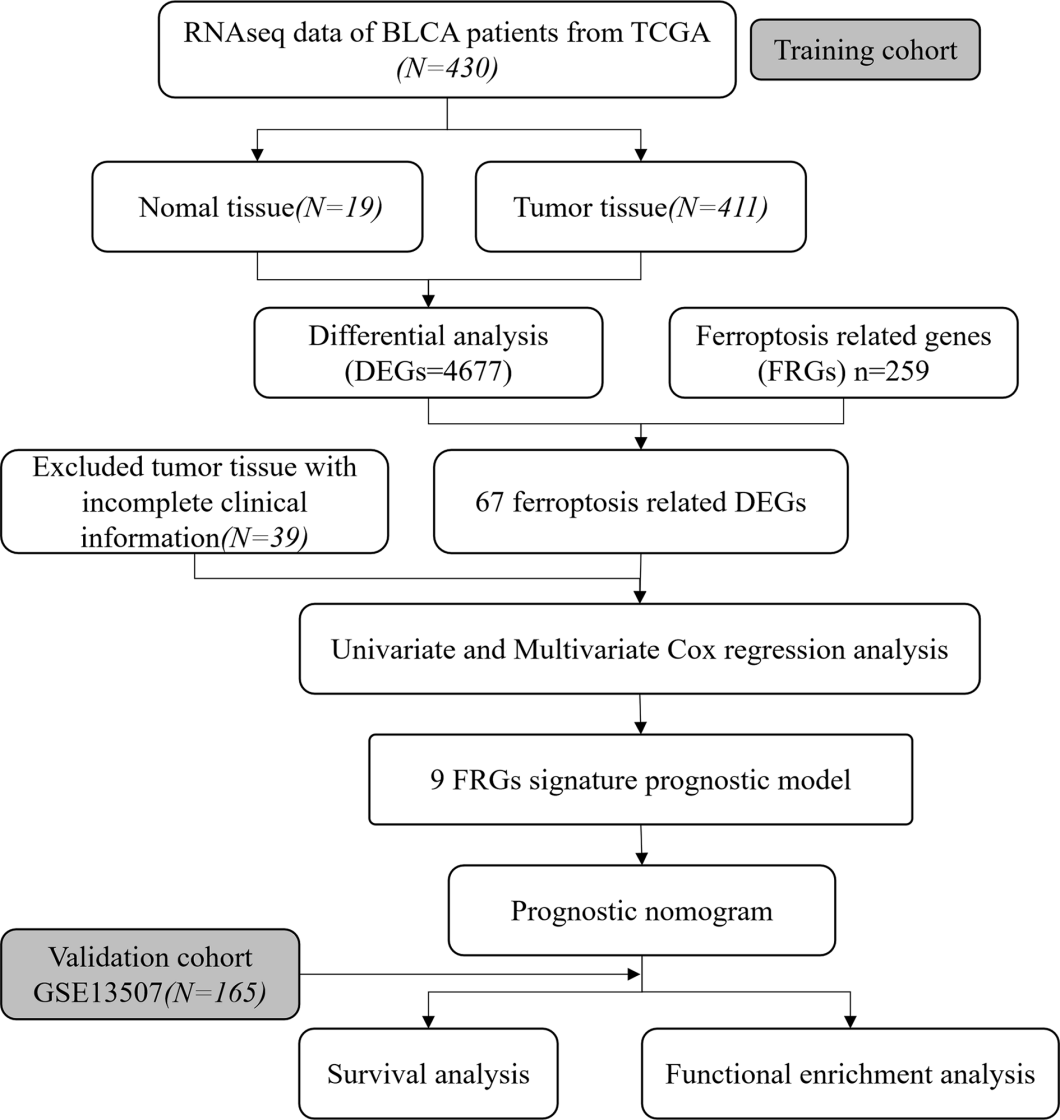
Flow chart of our study.

To systematically describe our study, the flow chart of the study is shown in **Figure 1**. From the TCGA RNA-seq dataset, we obtained expression data of 430 BLCA patients from the TCGA cohort and 165 patients from GEO (GSE13507). A total of 372 BLCA patients with complete clinical information were finally enrolled in the TCGA cohort. Baseline demographic and clinical characteristics of the included patents are shown in **Table 1**.

**Table 1:** Clinical characteristics of BLCA patients in the TCGA cohort and GEO GSE13507.

Characteristics		TCGA Total (*n* = 372)	GEO Total (*n* = 165)
**Age (years)**	<60	79 (21.2%)	42 (25.5%)
	≥60	293 (78.7%)	123 (74.5%)
**Gender**	Male	276 (74.2%)	135 (81.8%)
	Female	96 (25.8%)	30 (18.2%)
**T stage 1**	T0	1 (0.3%)	0
	T1	4 (1.1%)	80 (48.5%)
	T2	116 (31.2%)	31 (18.8%)
	T3	193 (51.9%)	19 (11.5%)
	T4	57 (15.3%)	11 (6.7%)
	Tx	1 (0.3%)	24 (14.5%)
**T stage 2**	T0–2	121 (32.5%)	111 (67.3%)
	T3–4	251 (67.5%)	30 (18.2%)
	Tx	1 (0.3%)	24 (14.5%)
**Lymph node metastasis**	Yes	124 (33.3%)	15 (9.1%)
	No	221 (59.4%)	149 (90.3%)
	Unknown	27 (7.3%)	1 (0.61%)
**Metastasis**	Yes	8 (2.2%)	7 (4.2%)
	No	183 (49.2%)	158 (95.8%)
	Unknown	181 (48.7%)	0
**Diagnosis subtype**	Papillary	117 (31.5%)	NA
	Non-papillary	250 (67.2%)	NA
	Unknown	5 (1.3%)	NA
**Histologic grade**	High	351 (94.4)	60 (36.4%)
	Low	19 (5.1%)	105 (63.6%)
	Unknown	2 (0.5%)	0
**Vital status**	Dead	166 (44.6%)	69 (41.8%)
	Alive	206 (55.4%)	96 (58.2%)

NA, Not Applicable.

### Identification of Prognostic DEGs Related to FRGs of BLCA in the TCGA Cohort

**Figure 2:**
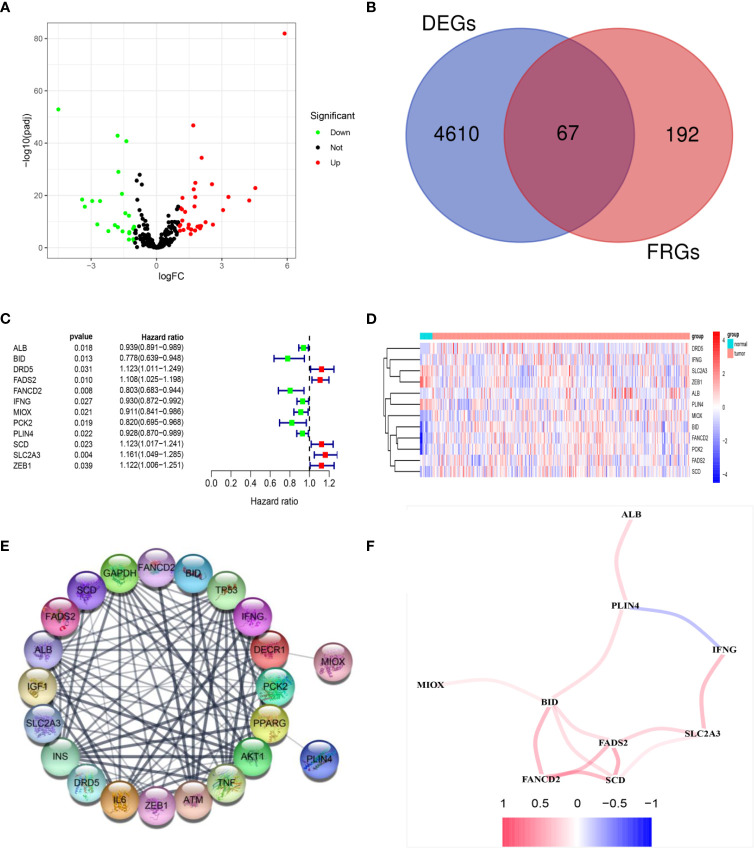
Identification of prognostic DEGs related to FRGs in the TCGA cohort. **(A)** The 67 overlapping genes were shown in the volcano map. Forty genes were upregulated and 27 genes were downregulated in tumor tissues. **(B)** Venn diagram to identify DEGs related to FRGs. **(C)** Univariate Cox proportional regression analysis showed that the 12 genes were significantly associated with OS. **(D)** A heatmap showing the expressions of the 12 prognostic genes in the tumors and normal tissues. **(E)** PPI network provided interactive information among the candidate prognostic genes. **(F)** The correlation network of candidate genes. Different colors represented the correlation coefficients.

The RNA expression data of 411 BLCA tumor samples and 19 adjacent normal samples were obtained from TCGA. Differential expression analysis was conducted with the DEseq2 package. A total of 4610 DEGs were screened out and a total of 67 FRGs (25.9%) were differentially expressed between tumor tissues and adjacent normal tissues (**Figures 2A, B**). Twelve candidate FRGs associated with OS were identified with univariate Cox regression analysis (**Figures 2C, D**). The protein–protein interaction network provided interactive information among these DEGs (**Figure 2E**). *BID*, *FADS2*, and *SCD* were identified as hub genes with “igraph” R package. The correlation network of these genes is presented in **Figure 2F**.

### Establishment of a Prognostic Model of FRGs in TCGA Cohort

**Figure 3:**
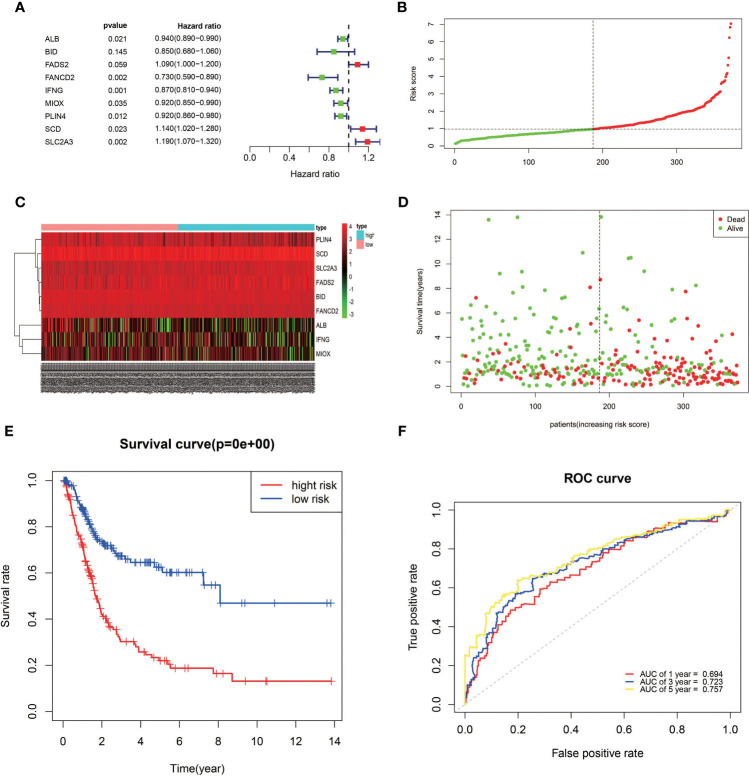
Establishment of a prognostic model of FRGs in the TCGA cohort. **(A)** A nine-gene signature was generated by Multivariate Cox regression analysis. **(B)** Distribution of signature-based risk scores. **(C)** The differences in the expression of the prognostic signature in different risk groups. **(D)** Survival status of high-risk and low-risk patients. **(E)** Kaplan–Meier curves indicated that the OS in the high-risk group was markedly poorer than that in the low-risk group (*p* < 0.0001). **(F)** Time-dependent ROC curve analysis for measuring the prognostic performance of the signature-based risk score on OS.

The expression profiles of the 12 candidate genes mentioned above were incorporated into a prognostic model using multivariate Cox regression analysis. A nine-gene signature, namely, *ALB, BID, FADS2, FANCD2, IFNG, MIOX, PLIN4, SCD*, and *SLC2A3*, was constructed (**Figure 3A**). Patients in the TCGA training cohort were classified as high risk (*n* = 186) or low risk (*n* = 186) based on the median cutoff value of risk score (**Figure 3B**). The risk score was calculated as follows: risk score= (−0.065* expression level of *ALB*) + (−0.165* expression level of *BID*) + (0.0898* expression level of *FADS2*) + (−0.3198* expression level of *FANCD2*) + (−0.14* expression level of *IFNG*) + (−0.085* expression level of *MIOX*) + (−0.087* expression level of PLIN4) + (0.1324* expression level of *SCD*) + (0.17* expression level of *SLC2A3*). The heatmap result showed high-risk group patients with higher expression levels of *FADS2*, *SCD*, and *SLC2A3* (**Figure 3C**). Patients in the high-risk group had a shorter survival time than those in the low-risk group (**Figure 3D**). Likewise, Kaplan–Meier survival curves show that OS of high-risk patients was significantly worse than OS of low-risk patients (**Figure 3E**). The predictive performance of the prognostic risk score model was evaluated by time-dependent ROC curves and the area under the curve (AUC). As shown in **Figure 3F**, the AUC reached 0.694 at 1 year, 0.723 at 3 years, and 0.757 at 5 years, suggesting a favorable predictive value of the risk score model in short- and long-term follow-up.

### Validation of the Prognostic Model based on Nine-FRG Signature in the GEO Cohort

**Figure 4:**
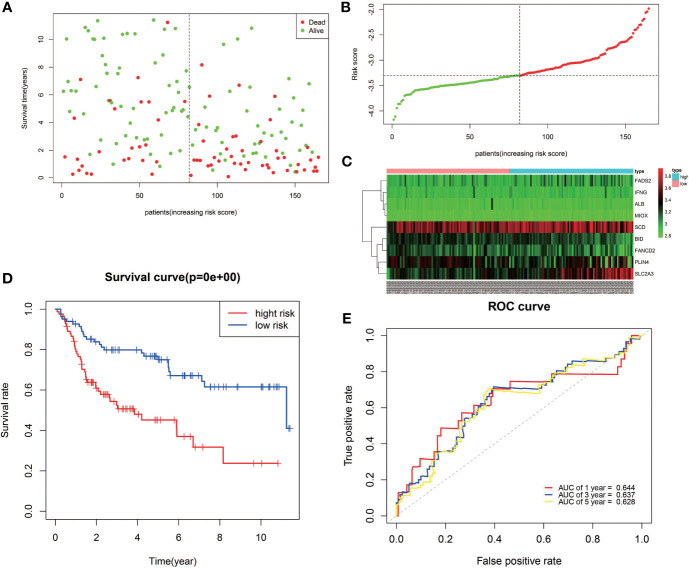
Validation of the nine-FRG signature in the GEO cohort. **(A)** Survival status of high-risk and low-risk patients. **(B)** Distribution of risk scores. **(C)** The differences in the expression of the prognostic signature in different risk groups. **(D)** Kaplan–Meier curves for OS. **(E)** Time-dependent ROC curve analysis.

The reliability of the model constructed from the TCGA cohort was validated in the GEO cohort. A total of 165 patients from the GEO cohort were divided into high-risk (*n* = 83) and low-risk (*n* = 82) groups by the median value calculated using the same risk formula and cutoff point obtained from the TCGA cohort (**Figure 4B**). The results are consistent with results obtained from the TCGA cohort. Patients in the high-risk group had a shorter survival time than those in the low-risk group (**Figure 4A**). Likewise, Kaplan–Meier survival curves show that OS of high-risk patients was significantly worse than OS of low-risk patients (**Figure 4D**). ROC curves also suggest a good predictive value of the risk score model (**Figure 4E**).

### Prognostic Analysis of the BLCA Patients Based on the Expression of the Nine-FRG Signature

**Figure 5:**
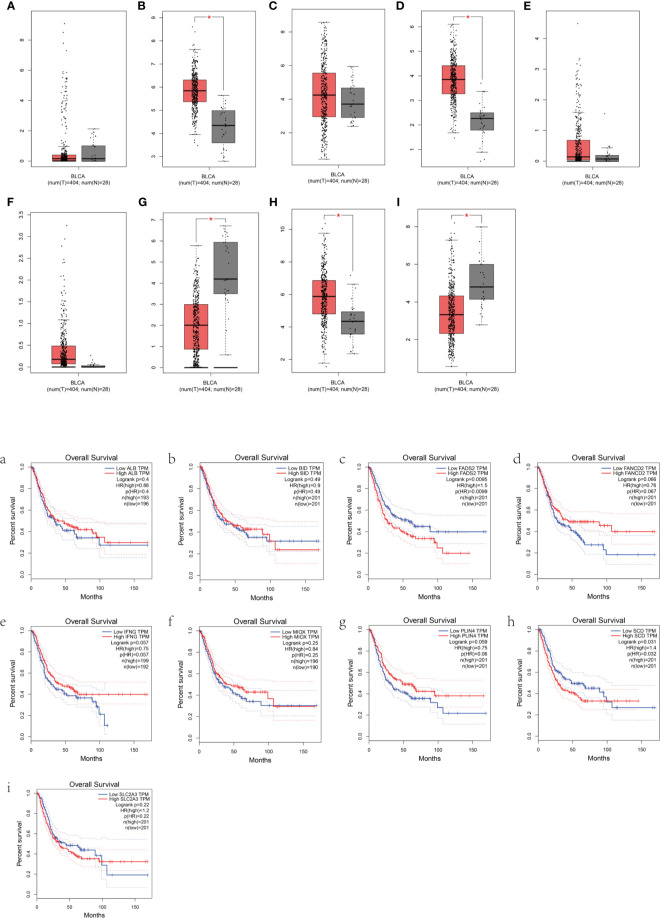
Prognostic analysis of the BLCA patients based on the expression of FRGs. **(A–I)** Box plots show the differences in the expression of nine different ferroptosis-related genes in the tumor and normal tissues from the GEPIA dataset. **(a–i)** The overall survival of BLCA patients based on the expression of the nine FRGs is shown.

To further determine the accuracy of the prognostic model of FRGs, the Gene Expression Profiling Interactive Analysis (GEPIA) database was used to analyze the OS of BLCA patients based on the expression of FRGs. Cutoff for high value and low value is set to 50%. *p* < 0.05 was considered statistically significant. In the signature genes, four genes, namely, *FADS2, SCD, IFNG*, and *PLIN4*, were significantly correlated with the OS of BLCA (**Figures 5C, E, G, H**). *FADS2* and *SCD* were unfavorable factors for OS of BLCA patients, whereas *IFNG* and *PLIN4* were favorable factors for OS of BLCA patients. This was consistent with results of multivariate Cox regression.

### Independent Prognostic Analysis of the Prognostic Model

**Figure 6:**
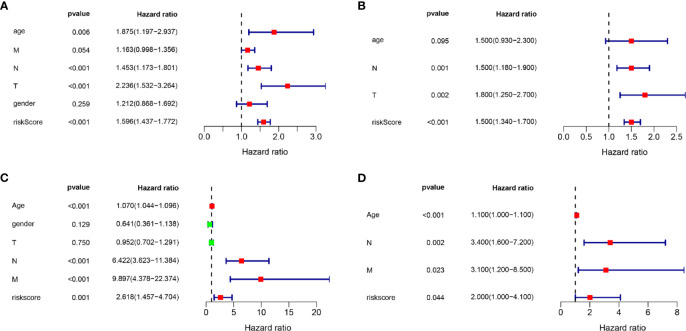
Univariate and multivariate Cox regression analyses. Results showing the signature-based risk score was an independent predictor of OS. **(A)** The univariate Cox regression analysis in the TCGA cohort. **(B)** The multivariate Cox regression analysis in the TCGA cohort. **(C)** The univariate Cox regression analysis in the GEO cohort. **(D)** The multivariate Cox regression analysis in the GEO cohort.

Univariate and multivariate Cox regression analyses were conducted to evaluate whether the signature-based risk score was an independent predictor of OS (**Figure 6**). Hazard ratios (HRs) and 95% confidence intervals (CIs) for each variable were calculated. *p* < 0.05 was considered statistically significant. In both TCGA and GEO data, results show that the risk scores were independent prognostic predictors for OS in the univariate and multivariate Cox regression analyses.

### Construction and Validation of the Nomogram in the TCGA Cohort

**Figure 7:**
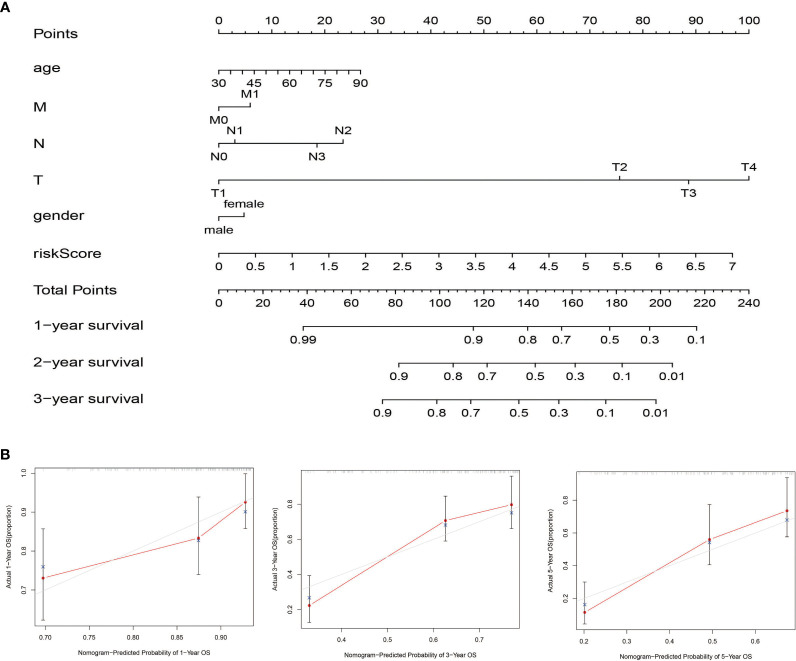
Construction and validation of the nomogram in the TCGA cohort. **(A)** The nomogram for predicting the OS of patients with BLCA at 1, 2, and 3 years in the TCGA dataset. **(B)** Calibration curves of the nomogram for OS prediction at 1, 3, and 5 years in the TCGA dataset.

Nomogram was generated based on several independent predictive factors to predict the probability of 1-year, 2-year, and 3-year OS rates with the TCGA Training dataset. Different factors were scored based on the proportion of contribution to survival risk as shown in **Figure 7A**. The calibration curve for the 1-year, 3-year, and 5-year OS probability results showed that the predicted survival rate is closely related to the actual survival rate (**Figure 7B**). These results indicated that the signature of the nine FRGs was a reliable prognostic indicator in BLCA patients.

### GO and KEGG Enrichment Analysis of the TCGA Cohort

**Figure 8:**
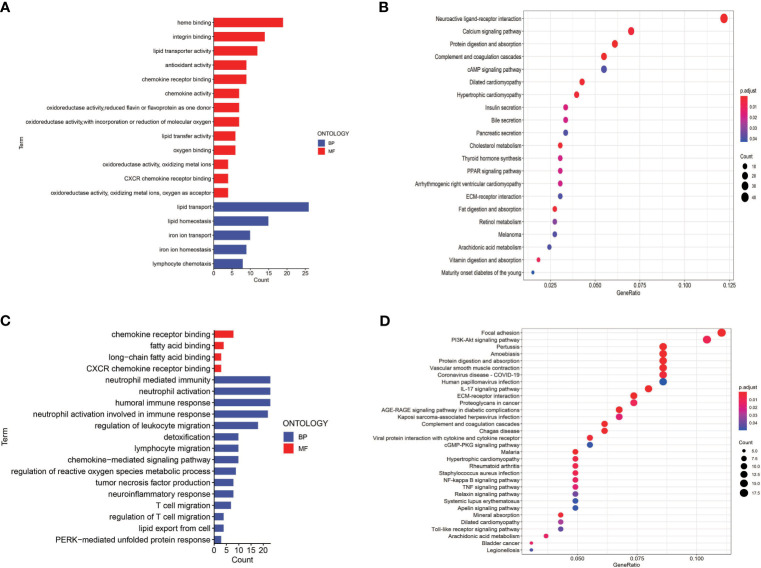
Gene Ontology (GO) and Kyoto Encyclopedia of Genes and Genomes (KEGG) enrichment analysis in TCGA and GEO cohorts. **(A)** GO enrichment analysis in the TCGA cohort. **(B)** KEGG enrichment analysis in the TCGA cohort. **(C)** GO enrichment analysis in the GEO cohort. **(D)** KEGG enrichment analysis in the GEO cohort.

To investigate the potential biological characteristics of the DEGs in high-risk and low-risk patients in the TGCA cohort, GO enrichment and KEGG pathway analyses were performed using the ClusterProfile R package. GO analysis indicated that DEGs were obviously enriched in some ferroptosis-related, immune-related biological processes and molecular functions (adjusted *p* < 0.05; **Figures 8A, C**). KEGG functional enrichment analysis suggested that DEGs were mostly enriched in the ferroptosis-related pathway, immune-related pathways, and bladder cancer (**Figures 8B, D**).

**Figure 9:**
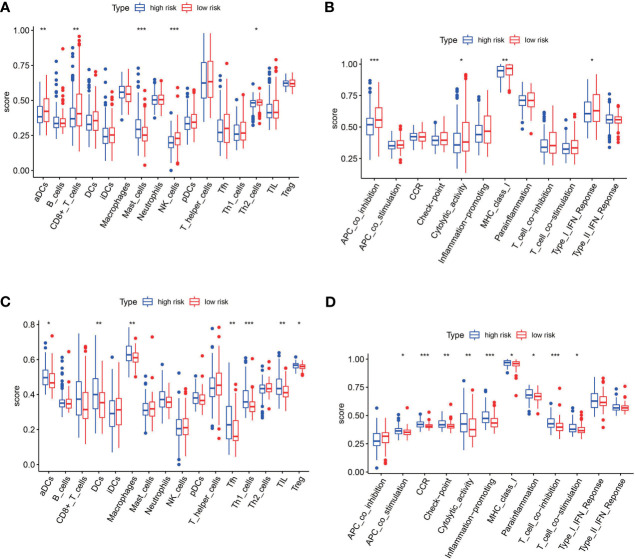
The single-sample gene set enrichment analysis (ssGSEA) scores between the high-risk and low-risk group in TCGA and GEO cohorts. **(A)** Box plots showing the scores of 16 immune cells in different groups in the TCGA cohort. **(B)** Box plots showing the scores of 13 immune-related functions in different groups in the TCGA cohort. **(C)** Box plots showing the scores of 16 immune cells in different groups in the GEO cohort. **(D)** Box plots showing the scores of 13 immune-related functions in different groups in the GEO cohort. Adjusted *p*-values are shown as follows: *p < 0.05; **p < 0.01; ***p < 0.001.

**Figure 10:**
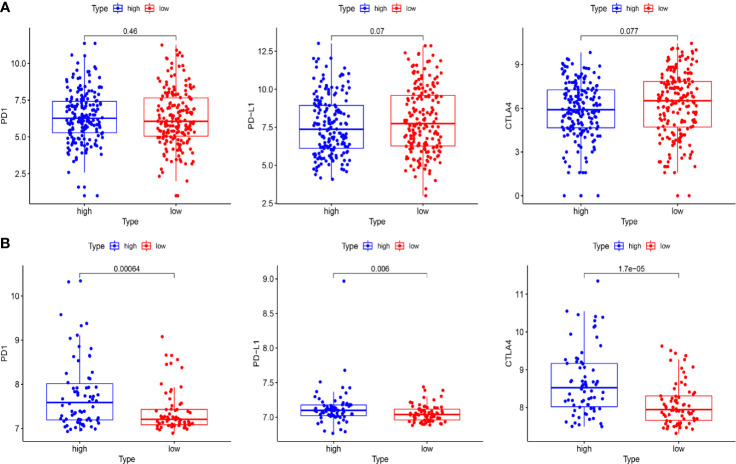
The expression of immune checkpoint molecules including PD1, PDL1, and CTLA4 between the high-risk and the low-risk group in TCGA and GEO cohorts. **(A)** Box plots show the differences in the expression of PD1, PDL1, and CTLA4 between the high-risk and low-risk group in the TCGA cohort. **(B)** Box plots show the differences in the expression of PD1, PDL1, and CTLA4 between the high-risk and the low-risk group in the GEO cohort.

To further explore the relationship between the risk score and immune status, we quantified the infiltrating scores of immune-cell and immunity-related pathways with ssGSEA. The correlations between ssGSEA scores and different risk groups showed that the scores of aDCs, mast cells, NK cells, APC co-inhibition, cytolytic activity, MHC class I, and type I IFN response were significantly different between the low-risk and high-risk groups in the TCGA cohort (**Figures 9A, B**). Interestingly, the scores of aDCs, DCs, macrophages, Tfh, Tfh1 cells, TIL, Treg, APC co-stimulation, CCR, checkpoint, cytolytic activity, inflammation promoting, MHC class I, parainflammation, T-cell co-inhibition, and T-cell co-stimulation were significantly different between the low-risk and high-risk groups in GEO cohorts (**Figures 9C, D**). Moreover, although the expression of immune checkpoint molecules including programmed cell death protein 1 (PD1), PD1 ligand 1 (PDL1), and cytotoxic T lymphocyte antigen 4 (CTLA4) was no statistical difference in TCGA cohort (**Figure 10A**), it significantly higher in the high-risk group in GEO cohort (**Figure 10B**).

## Discussion

Bladder cancer is a molecular heterogeneous malignant tumor; the treatment strategies for advanced-stage patients are limited. The molecular characteristics are closely related to prognosis of bladder cancer. Therefore, it is vital for improving the clinical outcome of BC patients to identify key biomarkers and targets affecting prognosis. The development of high-throughput array technology provides an opportunity to explore novel genes involved in the occurrence and development of BC. Increasing evidence has shown that ferroptosis plays a crucial role in tumorigenesis and cancer therapeutics (22). In this study, the differential expression of FRGs between BLCA tumor tissues and adjacent normal tissues were systematically investigated. FRGs associated with the prognosis of BLCA were determined by Cox proportional hazards regression analysis. Results significantly indicated the feasibility of building a prognostic model with these FRGs.

A novel prognostic model integrating nine ferroptosis-related DEGs was, for the first time, constructed and externally validated. These genes that make up the prognostic model were *ALB, BID, FADS2, FANCD2, IFNG, MIOX, PLIN4, SCD*, and *SLC2A3.* It was reported that *ALB* (albumin) may act synergistically with transferrin to limit iron supply, which may lead to the promotion of ferroptosis (23). The expression level of *ALB* was upregulated in BLCA tumor tissue compared with normal tissues (**Figure 5A**). The OS of the high-expression group was better than that of the low-expression group, which was consistent with the expression in the different risk groups based on the prognostic signature (**Figure 3C**). Ferroptosis is defined as an oxidative and iron-dependent pathway of regulated cell death, which is different from caspase-dependent apoptosis. Mitochondrial transactivation of *BID* links ferroptosis to mitochondrial damage as the last execution step of oxidative cell death (24). Overexpression of *BID* may promote the suppression of ferroptosis, indicating a worse prognosis (**Figure 5B**). *FADS2* is abnormally expressed in many malignant tumors, and its expression is significantly correlated with tumor proliferation, cell migration invasion, and ferroptosis (25). Activation of *FADS2* involved in the Warburg effect inhibits ferroptosis (26). Upregulation of *FADS2* was associated with poor prognosis in BLCA (**Figure 5C**). *SCD*, like *FADS2*, was involved in Warburg effect (26). A study (27) confirmed that *SCD* was an enzyme that catalyzes the rate-limiting step in monounsaturated fatty acid synthesis in ovarian cancer cells; inhibition of *SCD1* could induce both ferroptosis and apoptosis. *SCD* was highly expressed in ovarian cancer tissue. The expression levels of *SCD* in BLCA was also high (**Figure 5H**). *FANCD2*, a protein that mediates DNA repair, suppresses ferroptosis by transcription and transcription-independent mechanisms (28). *FANCD2* is closely correlated to tumorigenesis and progression (29). A study indicated that *FANCD2* expression correlated with the activation of apoptotic, cell cycle, and EMT pathways in clear cell renal cell carcinoma (30). The high expression level of *FANCD2* was related to better prognosis in BLCA (**Figure 4C**, **5D**), which suggests that the role of *FANCD2* in BLCA may be consistent with other studies. *IFNG* (interferon gamma, *INFγ*)released from CD8+ T cells downregulates the expression of *SLC3A2* and *SLC7A11*, two subunits of the glutamate-cystine antiporter system xc-, inhibits the uptake of cystine by tumor cells, and consequently promotes tumor cell lipid peroxidation and ferroptosis (31). Expression of *IFNG* was negatively associated, in BCLA patients, with patient outcome (**Figure 5E**). Overexpression of myo-inositol oxygenase (*MIOX*) exacerbates cellular redox injury in cisplatin-induced acute kidney injury (AKI) by accelerating ferroptosis (32). It is reasonable to assume that *MIOX* may play an anti-cancer role by promoting ferroptosis in BLCA. This could explain why *MIOX* is highly expressed in the low-risk group (**Figure 3C**). *PLIN4* (Perilipins4) is one of the families of lipid droplet-associated proteins that participate in lipid metabolism regulation. It can be used as diagnostic markers of liposarcoma and to differentiate liposarcoma subtypes (33). Compared with the corresponding normal tissues, the expression of *PLIN4* in BLCA tumor tissues was downregulated (**Figure 5G**), and higher expression of *PLIN4* was associated with better prognosis (**Figure 5G**). *PLIN4* could also be used as prognostic markers. Upregulation of the *SLC2A* gene that encodes the glucose transporter (GLUT) protein is associated with poor prognosis in many cancers. It was observed that upregulation of the *SLC2A3* genes is associated with decreased OS and DFS in colorectal cancer patients (34). Likewise, we found that *SLC2A3* expression was high in the high-risk group (**Figure 4C**). The nine FRGs were either positively or negatively correlated with ferroptosis. They were differentially expressed in different risk groups, which was consistent with their gene functions in cancers. However, not all nine genes had expression levels consistent with their functions in BLCA (**Figure 5**). Therefore, the specific role of these genes in BLCA has to be further investigated.

We further demonstrated that the risk score of the nine-gene signature was an independent prognostic indicator of OS for patients with BLCA. The high-risk group was found to have a significantly higher percentage of BLCA patients with worse clinicopathological features, such as an advanced T stage, lymph node metastasis, and histologic grade (**Table 2**). In addition, micropapillary carcinoma of the bladder (MPBC) is a variant type of infiltrating urothelial carcinoma, which portends a poor biological behavior in terms of disease stage at first diagnosis and clinical outcome (35). We tried to assess the risk of MPBC patients by our risk score, but unfortunately, the correlation between risk score and diagnosis subtype was not statistically significant. Thereafter, an individualized prognostic prediction model was constructed with nomograms, where the risks of individuals in the clinical context were quantified by integrating multiple risk factors including risk score. Calibration curves suggested high consistency between the actual and predicted OS rates. According to the aforementioned results, it was suggestive that the prognostic risk score model based on the nine-gene signature was a powerful prognostic indicator in BLCA patients.

**Table 2:** Baseline characteristics of the patients in different risk groups in the TCGA cohort and the GEO cohort.

Characteristics		TCGA cohort (*N* = 372)			GEO cohort (*N* = 165)		
		High risk	Low risk	*p*-value	High risk	Low risk	*p*-value
**Age (years)**				0.205			0.033
	<60	34	45		15	27	
	≥60	152	141		68	55	
**Gender**				0.075			0.84
	Male	130	146		67	68	
	Female	56	40		16	14	
**T stage**				<0.001			0.002
	T0–2	42	79		53	58	
	T3–4	143	107		23	7	
	Tx	1	0		7	17	
**Lymph node metastasis**				<0.001			0.243
	Yes	76	48		10	5	
	No	104	117		72	77	
	Unknown	6	21		1	0	
**Metastasis**				0.775			0.443
	Yes	5	3		5	2	
	No	91	92		78	80	
	Unknown	90	91		0	0	
**Diagnosis subtype**				0.075			
	Papillary	49	68		NA	NA	
	Non-papillary	135	115		NA	NA	
	Unknown	2	3		NA	NA	
**Histologic grade**				0.108			0.015
	High	180	171		38	22	
	Low	5	14		45	60	
	Unknown	1	1				

NA, Not Applicable.

To determine the role of ferroptosis-related classical signaling pathways in different risk groups, GO and KEGG enrichment analysis of DEGs in the high-risk and low-risk groups. Expectedly, the results indicated that DEGs were significantly enriched in biological oxidation, fatty acid metabolism, and iron metabolism (**Figure 5C**). These biological processes are all critical for the execution of ferroptosis (13, 17). Interestingly, many immunity-related functions and pathways were significantly enriched, such as chemokine receptor binding, humoral immune response, IL-17 signaling pathway, protein interaction with cytokine–cytokine receptor, and toil-like receptor signaling pathway (**Figures 8C, D**). ssGSEA revealed that the infiltrating scores of immune-cell and immunity-related pathways were significantly different in different risk groups. At present, many researchers have proven that ferroptosis is related to immunity. We found that NK cells, CD8+ T cells, and MHC class I molecules were significantly higher in the low-risk group (**Figures 9A, B**). A study indicated that ferritin heavy chain in tumor cells may modulate the expression of MHC class I molecules and influence NK cells (36). MHC class I molecules enable CD8+ T cells to recognize and kill tumor cells (37). CD8+ T cells release interferon (IFN)γ, and (IFN)γ can regulate the lipid peroxidation and ferroptosis pathways in tumors (31). In addition, studies have demonstrated that increased tumor-associated macrophages (38, 39) or Treg cells (39, 40) are related to poor prognosis in HCC patients due to their role in immune invasion. The fractions of macrophages and Treg cells were higher in high-risk group BLCA patients in the GEO cohort (**Figure 9C**), which were consistent with the abovementioned research results.

In recent years, immune checkpoint inhibitor treatment has become a new and promising therapy for BC. The recent IMvigor010 study (41) was designed to evaluate the role of a checkpoint inhibitor in muscle-invasive urothelial carcinoma (MIUC). Although the trial did not meet its primary endpoint of improved disease-free survival (DFS) in the atezolizumab group over observation because of higher frequencies of adverse events, we also could find that adjuvant checkpoint inhibitor therapy may have some advantages in muscle-invasive urothelial carcinoma. The stirring CheckMate274 study presented by Dean Bajorin at the 2021 ASCO Genitourinary Cancers Symposium indicated that the adjuvant nivolumab, a PD-1 immune checkpoint inhibitor, significantly improved DFS in patients with high-risk MIUC after radical surgery, especially in PD-L1≥1% patients. There was significant difference in checkpoint between the high-risk and low-risk patients in our study (**Figure 9D**). The expression of immune checkpoint molecules including PD1, PDL1, and CTLA4 was significantly higher in the high-risk group in GEO cohorts (**Figure 10B**). This indicates that patients in the high-risk group may benefit more from immune checkpoint inhibitor therapy than patients in the low-risk group and provides new insight for BC immunotherapy. Considered together, these findings suggest that poor prognosis of patients with high risk might be correlated with immunosuppression, and ferroptosis could play a role in the immunotherapy of BC.

Despite the confirmation of our prognostic model in various datasets, this study was limited because it was a retrospective study. A further well-designed prospective study is necessary to validate the clinical value of the developed model. Besides, it was inevitable that by merely considering a single hallmark to build a prognostic model, many prominent prognostic genes in BC might have been excluded.

In conclusion, a novel prognostic model based on the nine-FRG signature in BLCA was the first established and validated. The prognostic models exhibited superior predictive performance and could independently predict the prognosis of BC patients. Understanding the roles of the signature and the relationship between ferroptosis and tumor immunity can provide insights into prognostic and therapeutic implications for BC patients.

## Data Availability Statement

The datasets presented in this study can be found in online repositories. The names of the repository/repositories and accession number(s) can be found in the article/**Supplementary Material**.

## Ethics Statement

Ethical review and approval was not required for the study on human participants in accordance with the local legislation and institutional requirements. Written informed consent for participation was not required for this study in accordance with the national legislation and the institutional requirements.

## Author Contributions

YY and YH conceived and designed the study. LY and CL provided equal contributions to research design, data analysis and article writing. YQ, GZ, BZ, and ZW revised the manuscript. All authors contributed to the article and approved the submitted version.

## Funding

This study was supported by Joint Special Fund for Applied Basic Research of Yunnan Provincial Science and Technology Department-Kunming Medical University (2019FE001(-087) and Innovation Fund for Postgraduates of Kunming Medical University (2021D11).

## Conflict of Interest

The authors declare that the research was conducted in the absence of any commercial or financial relationships that could be construed as a potential conflict of interest.

## Publisher’s Note

All claims expressed in this article are solely those of the authors and do not necessarily represent those of their affiliated organizations, or those of the publisher, the editors and the reviewers. Any product that may be evaluated in this article, or claim that may be made by its manufacturer, is not guaranteed or endorsed by the publisher.
